# Can the combination of internal iliac temporary occlusion and uterine artery embolization reduce bleeding and the need for intraoperative blood transfusion in cases of invasive placentation?

**DOI:** 10.6061/clinics/2019/e946

**Published:** 2019-06-11

**Authors:** Salomão Faroj Chodraui-Filho, Lucas Moretti Monsignore, Rafael Kiyuze Freitas, Guilherme Seizem Nakiri, Ricardo de Carvalho Cavalli, Geraldo Duarte, Daniel Giansante Abud

**Affiliations:** I Divisao de Radiologia Intervencionista, Departamento de Imagens Medicas, Hematologia e Oncologia Clinica, Faculdade de Medicina de Ribeirao Preto, Universidade de Sao Paulo, Ribeirao Preto, SP, BR; II Divisao de Obstetricia, Departamento de Ginecologia e Obstetricia, Faculdade de Medicina de Ribeirao Preto, Universidade de Sao Paulo, Ribeirao Preto, SP, BR

**Keywords:** Abnormally Invasive Placenta, High-Risk Pregnancy, Postpartum Hemorrhage, Embolization, *Placenta Accreta*

## Abstract

**OBJECTIVES:**

Women with invasive placentation (IP) are at high risk of life-threatening hemorrhage. In the last two decades, less invasive surgical approaches combined with endovascular procedures have proven to be safe. Most case series describe the use of temporary balloon occlusion and embolization, either combined or not. Concerning hemorrhage rates, each separate interventional approach performs better than surgery alone does, yet it is not clear whether the combination of multiple interventional techniques can be beneficial and promote a lower incidence of intrapartum bleeding. We aim to evaluate whether combining temporary balloon occlusion of the internal iliac artery and uterine artery embolization promotes better hemorrhage control than do other individual interventional approaches reported in the scientific literature in the context of cesarean birth followed by hysterectomy in patients with IP.

**METHODS:**

This is a retrospective analysis of patients with confirmed IP who underwent temporary balloon occlusion and embolization of the internal iliac arteries followed by puerperal hysterectomy. We compared patient results to data extracted from a recent systematic review and meta-analysis of the current literature that focused on interventional procedures in patients with IP.

**RESULTS:**

A total of 35 patients underwent the procedure during the study period in our institution. The mean volume of packed red blood cells and the estimated blood loss were 487.9 mL and 1193 mL, respectively. Four patients experienced complications that were attributed to the endovascular procedure.

**CONCLUSION:**

The combination of temporary balloon occlusion and uterine artery embolization does not seem to promote better hemorrhage control than each procedure performed individually does.

## INTRODUCTION

Failed placental detachment is the leading cause of third trimester bleeding and complications that lead to maternal death 1,2. Women with invasive placentation (IP) are at high risk of life-threatening hemorrhage, with reports of maternal mortality as high as 7% 3,4. Although IP is a somewhat uncommon condition 5, we have noticed a rising incidence of IP alongside the increasing number of surgical deliveries worldwide 6-10.

The average blood loss during birth among women with IP is 3000-5000 mL, and thus, transfusion is required in as many as 90% of patients 1,4,11. When facing IP, the recommendation for primary treatment remains a planned cesarean hysterectomy when IP is suspected prenatally 3,9. However, there is a notable increase in the literature aiming for uterine preservation with less invasive approaches combined with endovascular procedures.

A few different interventional radiology (IR) techniques have been reported since Dubois first reported a case series in which balloon occlusion and embolization of the internal iliac arteries (IIA) were used to prevent postpartum hemorrhage 12. Most case series describe the use of temporary balloon occlusion of different levels of the abdominopelvic circulation, such as the distal aorta, common iliac artery and IIA associated or not with pelvic embolization. The results vary between the different approaches; however, they demonstrate that endovascular interventions have noticeable value in reducing hemorrhage in abnormal placentation births 13. Concerning hemorrhage rates, each interventional approach performs better than surgery alone does, yet it is not clear whether the combination of multiple interventional techniques can be beneficial and promote reduced intrapartum bleeding in the context of this disease.

We present a single-center retrospective study in which temporary prophylactic balloon occlusion of the IIA followed by bilateral uterine artery embolization (PBOIIA+UAE) was used to aid puerperal hysterectomy aimed at reducing bleeding and transfusion needs in patients with histopathologically confirmed IP.

In this study, we aim to evaluate whether combining temporary balloon occlusion of the IIA and embolization of the uterine artery result in better outcomes than do other individual interventional approaches reported in the scientific literature in the context of cesarean birth followed by hysterectomy in patients with IP.

## MATERIALS AND METHODS

We conducted a retrospective study of all patients who underwent PBOIIA+UAE and puerperal hysterectomy for IP treatment from January 2012 to November 2016. The selected patients selected were those with a high clinical suspicion of IP and signs of IP on magnetic resonance imaging or ultrasound. Patients without histopathologically confirmed IP and those who did not receive embolization were excluded from the evaluation. The obtained patient data include a detailed gestational and previous surgical history, a log of transfused blood products, a record of hemoglobin levels throughout the procedure, documentation of the duration of hospital stay and details of any procedure-related complications.

The histopathological reports were analyzed and categorized according to the classical definition of IP 7. *Placenta accreta* was characterized as trophoblastic invasion beyond the decidua basalis without muscular involvement. *Placenta increta* was diagnosed if there were signs of deep myometrial invasion, and if there were signs of serosa breach, the status of *placenta percreta* was assigned. The subtypes are generally referred together as IP.

Both the endovascular and surgical procedures were performed in the same operating room. Patients underwent cystoscopy the day before delivery or immediately before surgery; ureteral catheters were placed if signs of vesical or parametrium invasion were observed or suspected. Attending anesthesiologists chose between general anesthesia or spinal block and sedation.

**Figure 1:**
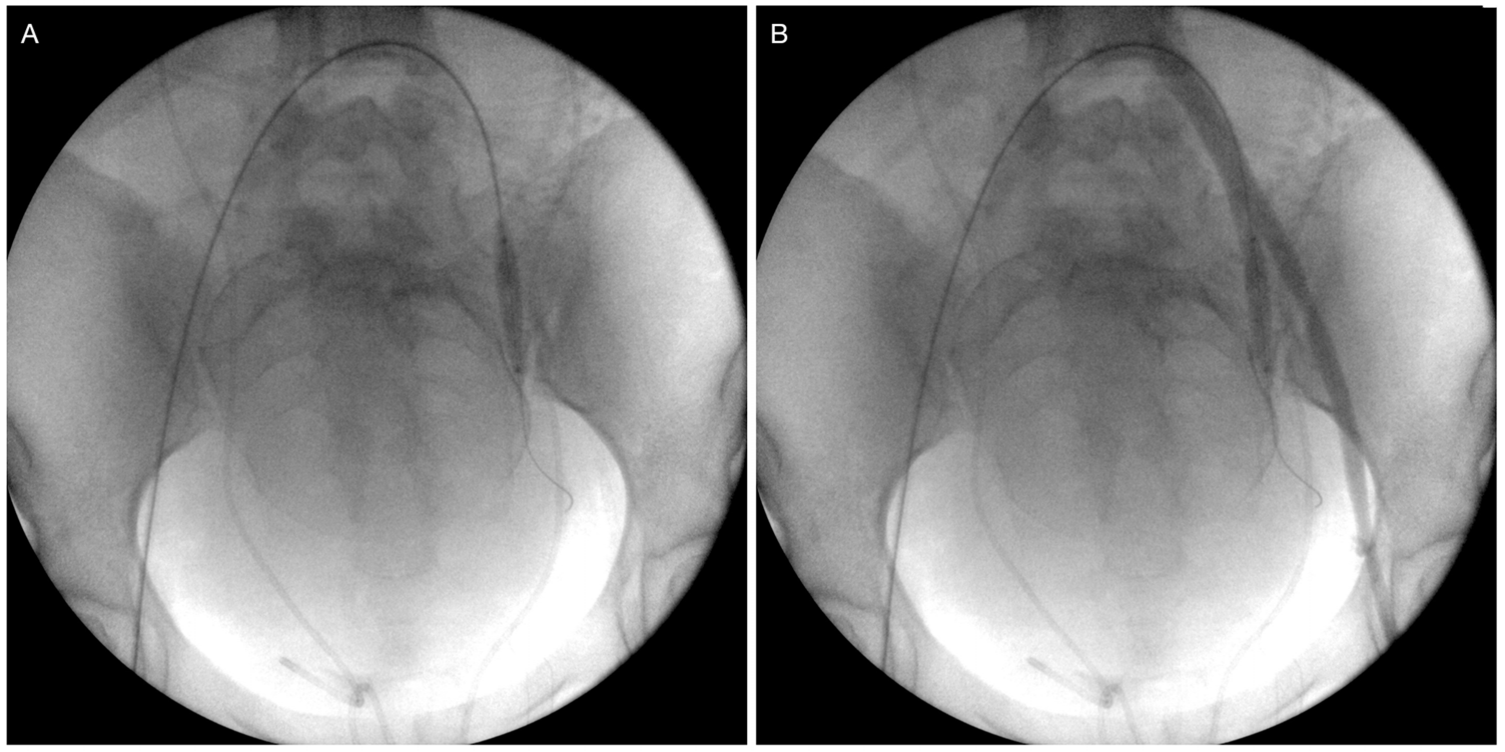
Balloon catheter placement and testing. (A) Inflated balloon catheter positioned in the left internal iliac artery. (B) Retrograde contrast injection through the left femoral sheath confirming the correct placement of the balloon and the absence of flow distally into the internal iliac branches.

We used the bilateral retrograde transfemoral approach to place a 7-French sheath via the Seldinger technique. Pressurized saline solution was used to continuously irrigate both sheaths. Under fluoroscopic guidance and using anatomical parameters, noncompliant balloon catheters (Mustang, Boston Scientific, Marlborough, MA, USA; Advance 35LP, Cook Medical, Bloomington, USA) were placed in both IIA over a 0.035-in, 260-cm angled stiff hydrophilic guide wire (Radiofocus, Terumo, Tokyo, Japan) after selective catheterization using a Cobra 2 5-French angiographic catheter (Performa, Merit Medical, South Jordan, USA; Optitorque, Terumo, Japan). Balloon catheter sizes ranged from 20 to 40 mm in length and from 8 to 9 mm in diameter. After balloon inflation with the minimum volume that was sufficient to block blood flow, we performed small-volume contrast injections through the sheaths to test and confirm artery occlusion (Figure 1). Cesarean delivery was performed via the vertical abdominal incision approach. Immediately after birth, the balloons were inflated to the previously tested volumes. The placenta was kept in place, the uterine wall was sutured and the ovarian anastomosis was ligated.

**Figure 2:**
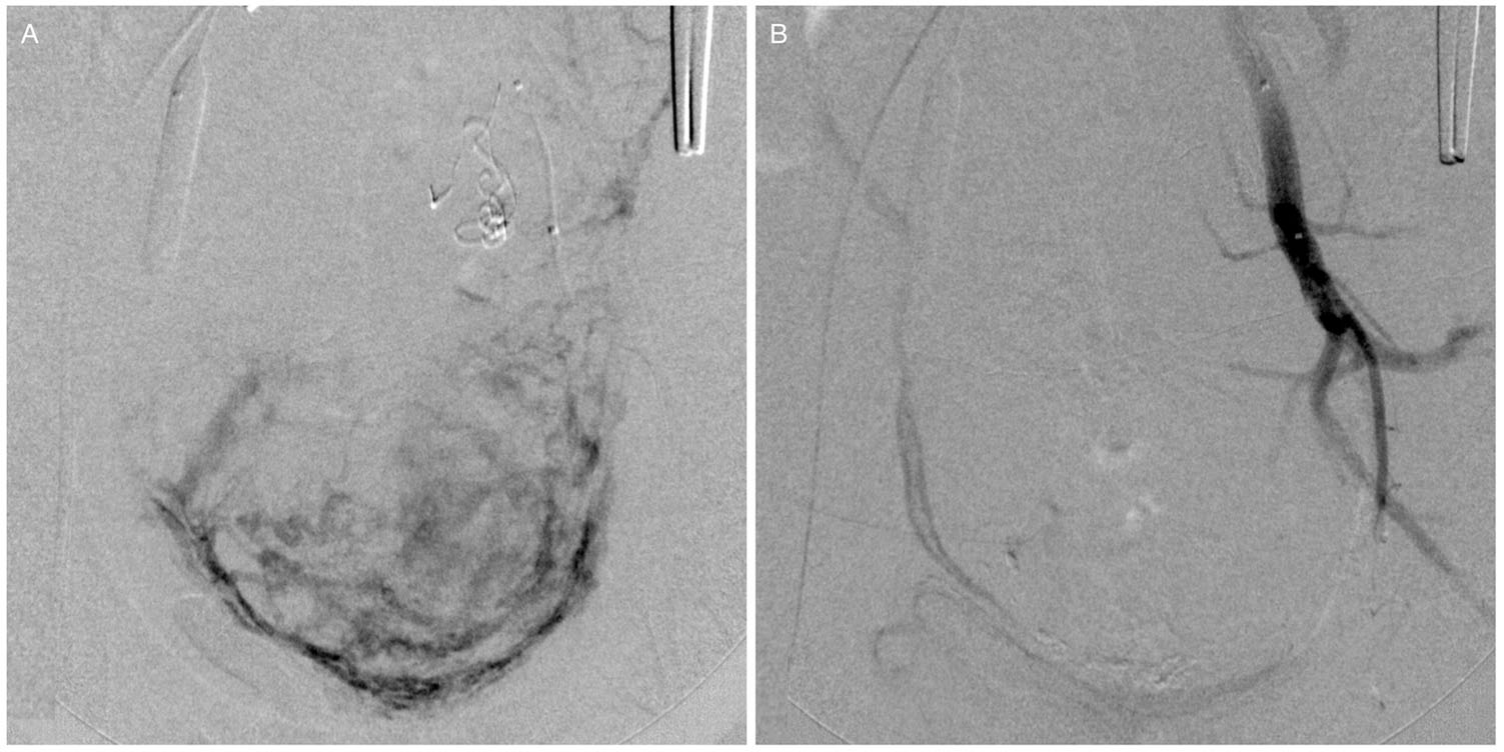
(A) Angiographic images obtained before embolization depicting the enhancement of the uterine vessels. (B) Final angiographic results after embolization. Note the absence of enhancement in the uterine territory and the flow arrest at the left internal iliac artery branches.

**Figure 3:**
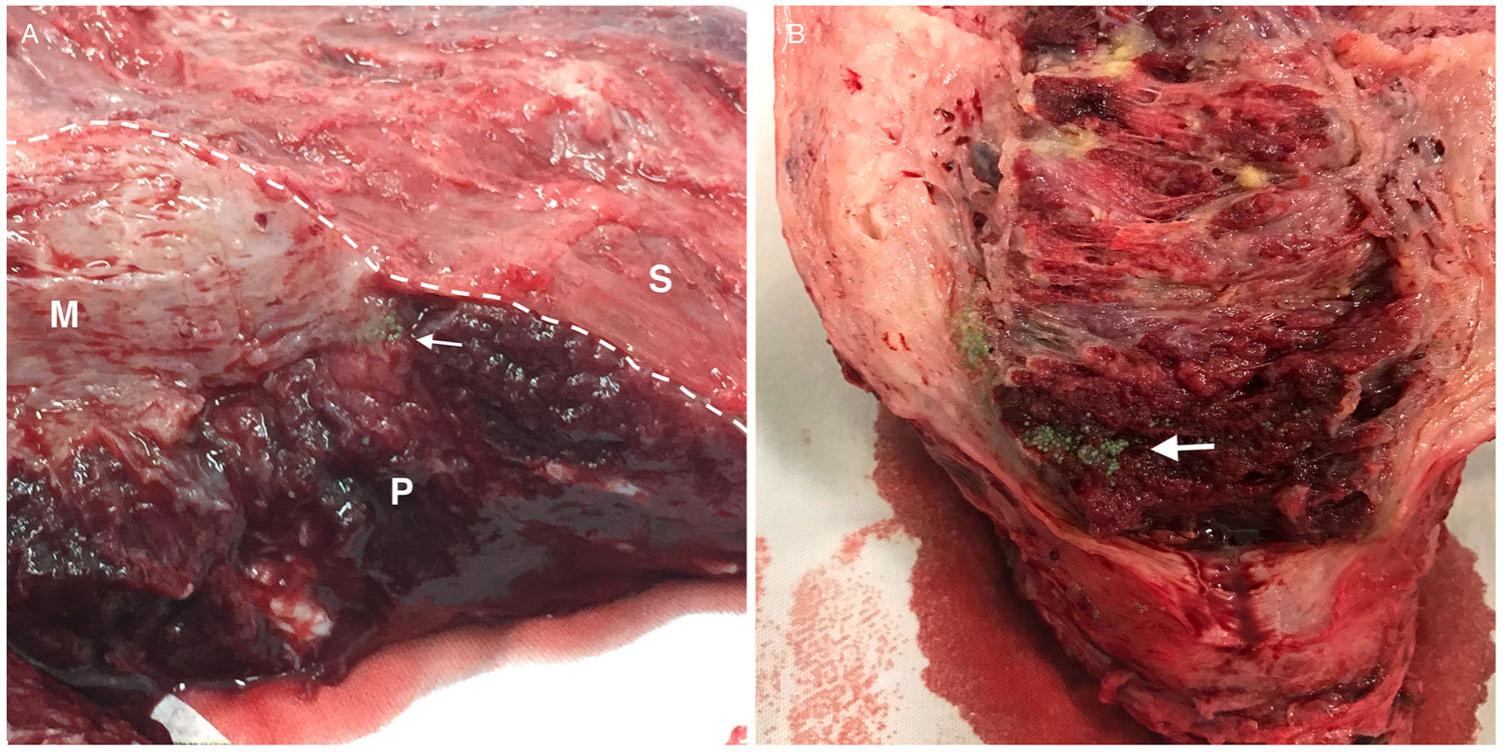
(A, B) Hysterectomy specimens submitted to postoperative posterior longitudinal hysterotomy. Embolic microspheres (arrows) deep in the placental tissue (P). Note the narrowing of the myometrium (M) in the lower uterine segment where there is contact between the placenta and the uterine serosa (S), which is a typical characteristic of *placenta percreta*.

The second stage of the endovascular procedure began with acquiring a series of angiographic images of each balloon catheter to assess the origin of the uterine arteries. The same balloon catheters were used for embolization. When the uterine artery (UA) was successfully catheterized, embolic microspheres (700-900 or 900-1200 µm, Embospheres, Biosphere Medical, Paris, France; 700 or 900 µm, Embozene, CeloNova BioSciences, San Antonio, TX, USA; or 700-900 or 900-1200 µm, BeadBlock, Biocompatibles, Farnham, United Kingdom) diluted with a 50% strength iodine contrast solution were injected under fluoroscopic guidance until flow reduction was achieved. A mixture of 2-mm pieces of absorbable gelatin sponge (Gelfoam, Pharmacia & Upjohn Co., Kalamazoo, MI, USA) and 50% strength iodine contrast was injected until the uterine arterial flow was completely blocked. When the uterine artery was not selectively catheterized, we first used small, 2-mm pieces of absorbable gelatin sponge to embolize the IIA. Once flow arrest or retardation to the muscular branches and flow redirection to the uterine artery were observed, we used microspheres to achieve satisfactory flow reduction of the uterine artery and then performed embolization of the IIA with absorbable gelatin sponges. Embolization was considered complete when flow arrest was evident with the balloons deflated (Figure 2). Inflation of the balloons was maintained while the obstetricians proceeded with the hysterectomy (Figure 3). The balloons were then deflated and removed, followed by the removal of the sheaths and manual compression of the puncture sites.

Calculated blood loss (CBL) among patients who did not receive transfusion products was calculated using an adaptation of the Meunier's hemoglobin dilution formula 14. Among patients who underwent transfusion, we adjusted the hemoglobin levels to the amount of red blood cells transfused based on findings by Elzik et al. 15.

### Ethical approval

The institutional review board approved the study with a waiver for a consent form issued by the “Comitê de Ética em Pesquisa do Hospital das Clínicas da Faculdade de Medicina de Ribeirão Preto da Universidade de São Paulo, Brasil”, under the number 1.908.096, approved on February 06, 2017.

## RESULTS

Thirty-five patients underwent PBOIIA+UAE and puerperal hysterectomy for IP treatment at our institution from 2012 to 2016 (Table 1). The mean age was 33 years old (range 24 to 43 years old). Only 3 patients did not undergo previous cesarean sections but presented a history of uterine surgical manipulation (uterine curettage – patient numbers 1 and 35; myomectomy – patient number 7). Among the remaining 32 patients, the mean number of previous cesarean sections was 2.4, ranging from 1 to 4. The mean gestational age at birth was 33 weeks and 6 days, ranging from 25 weeks and 6 days to 38 weeks and 2 days. Histopathological reports placed 25.7% (9/35) of patients in the *accreta* category, 31.4% (11/35) in the *increta* category and 42.8% (15/35) in the *percreta* category.

The mean global CBL was 1193 mL (range 157 to 2967 mL; SD 679 mL), with lower CBL in patients with the *accreta* subtype than in patients with the *increta* and *percreta* subtypes (Table 2). Hemoglobin levels prior to the procedures ranged from 9.8 mg/dL to 14.3 mg/dL (mean 11.53 mg/dL). Eighteen patients (51.4%) did not require any type of transfusion to maintain stability. The volume of transfused RBCs in the remaining 17 patients ranged from 278 mL to 3384 mL (mean 1004 mL; SD 738 mL).

There was no maternal mortality during the procedures or during the follow-up period. One case of fetal death was reported due to chromosomal disease not compatible with extrauterine life. Hospital records revealed complications in the cases of 9 patients (21.2%), among which only 4 cases (11.2%) of complications were attributed to the endovascular procedure itself. Of those, two were diagnosed with acute arterial thrombosis, one with gluteal muscle necrosis and one with minor subcutaneous and skin lesions. No anatomical or technical discrepancies were identified in the patients who experienced necrosis. In both cases, initial embolization from the uterine arteries was performed using 700-900 µm embolic microspheres, which was the most frequently utilized size in our cohort (40% of the cases). Procedure length and CBL were also between average, and in both cases, there were no signs of extrauterine invasion. No additional interventions were needed to treat the patient with skin lesions, and follow-up revealed full recovery in three weeks. Three patients showed signs of minor surgical wound infection, and all of them were successfully treated with antibiotics. Two patients underwent unilateral ureteral reconstruction, and one patient underwent bladder reconstruction at the time of hysterectomy. One patient presented with vaginal bleeding secondary to a small vaginal cuff tear, which was promptly surgically assessed and repaired.

The procedure duration was evaluated in 34 patients (one patient was missing this information in her medical records), and the procedure length ranged from 200 minutes to 475 minutes (average 332 minutes, SD 70 minutes), with no significant difference among the histopathological subtypes of IP (*p*=0.642). The length of hospital stay ranged from 2 to 22 days (mean 7.7 days, SD 5.3 days), with no significant differences among the histopathological subtypes (*p*=0.138). Only 10 patients stayed longer than one week, three of whom waited for their newborns to be discharged, two for the treatment of surgical wound infections and one for a follow up of the ureteral reconstruction surgery. One patient presented with signs of puerperal psychosis, and one patient presented with psychological grieving and received counselling that extended her hospital stay. No data are presented regarding radiation exposure in this series of patients.

## DISCUSSION

Due to the recent increase in the incidence of and the ability to prenatally diagnose IP, discussions involving alternatives for the prevention of hemorrhage in patients with IP have multiplied. Many IR procedures are available to aid cesarean birth in patients with IP. Reports on prophylactic balloon occlusion of the abdominal aorta (PBOAA), of the common iliac arteries (PBOCIA) and of the internal iliac arteries (PBOIIA) and uterine artery embolization (UAE) followed or not by hysterectomy have been described and shown to prevent intrapartum hemorrhage in patients with IP when compared to hysterectomy alone 16-18. Most of the data available come from retrospective series and few randomized studies; thus, there are heterogeneous results regarding the efficiency and safety of each approach.

The present series reports the use of PBOIIA+UAE and puerperal hysterectomy for IP. Contrary to more recent trends in publications, in which the primary aim was to avoid hysterectomy, immediate postpartum hysterectomy was defined as the standard of care at our institution. Data regarding the effectiveness of PBOIIA to prevent the need for hysterectomy remain controversial 5,19,20.

A recent systematic review published by Shahin and Pang 13 compared 69 studies involving 1395 patients undergoing different approaches aimed to assist birth in the context of abnormal placentation. Our case series revealed mean blood loss rates (1193 mL [range 157 to 2967 mL; SD 679 mL]) that are compatible with those indicated in the previous review (Table 3). The same review showed that in a subgroup analysis of PBOIIA for caesarean section and caesarean hysterectomy, only the latter had a positive impact on blood loss. The key points revealed by Shahin and Pang's study are that the balloon positioning seems to have an impact on hemorrhage control and complication rates and suggests that positioning the balloons at the abdominal aorta might be the most effective protection against bleeding, whereas distal positioning of the balloon (as in PBOCIA or PBOIIA) may increase the rates of thromboembolic complications.

During regular cesarean sections, the estimated blood loss (EBL) is approximately 1000 mL, and a blood transfusion is usually not required 21. Among the patients with IP, the EBL during deliveries without the incorporation of IR procedures to minimize bleeding varies from 3000 to 5000 mL, and transfusion is required in up to 95% of patients 4. In deliveries assisted with IR techniques, the mean EBL ranges from 586 to 15000 mL 13. These heterogeneous results depend on multiple factors, for instance, whether the placenta was left in place, if hysterectomy was performed, variations in the degree of placental invasion and variations in experience of the multidisciplinary surgical team. Although cesarean birth followed by hysterectomy presents higher EBL rates than purely cesarean birth does, postoperative bleeding complications are far more common during conservative uterine management, affecting up to 13.5% of patients 22,23. As expected, higher bleeding reports and transfusion needs are observed in patients with *placenta percreta* and *incret*a than in those with *placenta accreta* 22. In our patient cohort, the mean CBL was 1193 mL, with a significantly lower mean CBL in patients with the IP classification (706.5 mL). This number is expressively smaller than that observed in reports of patients who underwent hysterectomy alone (1000-4446 mL) 13.

More than half of our patients did not require transfusion, agreeing with the results reported in Shahin and Pang's meta-analysis, which revealed a total of 11 studies in which patients who underwent endovascular intervention had lower RBC unit transfusion needs than did patients who did not receive endovascular intervention. In our series, the average global procedure time was 332 minutes. This considerable procedure duration is directly related to the intent of hysterectomy and the fact that the records accounted for the entire time the patient was in the OR, including the time for placing ureteral catheters and initiating anesthesia and not just the time required for the interventional procedures themselves.

The balloon-related complications described in the literature vary from 3 to 50% and include groin hematoma 24-26; fetal bradycardia 25; arterial thrombosis and limb ischemia 17,24,27,28; and iliac dissection 27. The present series reports complications in 11% of cases, including two cases of femoral thromboses, one of gluteal muscular necrosis and one of subcutaneous and skin lesions. However, Shahin and Pang's meta-analysis revealed a total of 16 occurrences of lower limb arterial thromboses (1.14% of the 1485 patients submitted to all interventions), which was significantly lower than the incidence of lower limb arterial thromboses reported in our series (5.7%), especially if we take into consideration that the review accounted for studies in which large-bore sheaths were used for the PBOAA technique. As a note, we state that the use of angioplasty balloons, instead of typical occlusion-type compliant balloons, is a result of the equipment availability in our institution. The most comprehensive literature to date suggests that the arterial thrombosis complications observed in our cohort may be related to the positioning of the balloons in the IIA. Since both hemorrhage control and complication rates were compatible with those observed in the literature for other techniques alone, this finding raises the discussion of whether the use of the combined approach is indeed beneficial, when applied distinctively in cases of low-stage wall invasion 29,30.

Embolization-related complications vary from 8 to 36% and include groin hematoma 31, skin necrosis 28, fetal death 32, hematochezia 33, ovarian vein thrombosis 33, uterine synechiae 33, endometrial atrophy 33 and endometritis 34; the latter ones apply to cases in which the uterus was left in place and is not suitable for comparison to the cases in our study. Once again, Shahin and Pang's meta-analysis revealed 10 patients who underwent PBOIIA or PBOCIA and developed intermittent lower limb or buttock claudication but found no reports of actual necrosis in the follow up.

The current study has a few limitations, most of which are intrinsic to the retrospective design. Blood loss estimations had to be calculated using an indirect method, and although the hemoglobin dilution method is imperfect, it can be used as an estimate 14. It is worth mentioning that the methods used for calculating intrapartum bleeding have not been mentioned in most papers regarding the use of IR techniques for the treatment of IP. In our series, all procedures took place away from the Interventional Radiology department, using mobile C-arms at the institution's surgical unit, where no routine protocol to register the radiation dosages was previously established. Although we lack radiation exposure records, we believe that dosages did not exceed those encountered during similarly described techniques; therefore, we took into consideration that the fetuses were exposed to fluoroscopy only during the positioning of the balloon catheters in both IIA and that no actual angiographic series where performed before birth. Since all patients underwent hysterectomy, the impact of radiation on fertility is not a concern among this patient population.

In summary, PBOIIA+UAE followed by hysterectomy seems to be feasible and to effectively reduce blood loss and transfusion requirements among this specific group of patients, yet with few remarkable contributions toward improving patient outcomes. Since the combination of the two techniques does not seem to perform better than single-technique approaches do, and our cohort of patients presented a higher complication rate than expected, the study suggests that using a single interventional technique may be preferred. As expected, because most data available to date come from small series and nonrandomized studies, the need for prospective randomized controlled trials evaluating the performance of all interventional techniques in patients with IP remains.

## AUTHOR CONTRIBUTIONS

Chodraui-Filho SF, Monsignore LM, Freitas RK and Nakiri GS have participated in data collecting, manuscript writing and editing, and performing the procedures. Cavalli RC, Duarte G and Abud DG contributed to consulting, writing and editing the manuscript and writing and performing the procedures described in the manuscript.

## Figures and Tables

**Table 1:** Individual clinical characteristics, transfusion needs and birth-related morbidity.

Number	Age (years)	Gestational age	Previous cesarean sections	Histological subtype	Volume of RBCs transfused (mL)	CBL (mL)	Endovascular-related complications	Birth-related complications	Hospital stay (days)
1	38	28w	0	*Increta*	1513	2116	AAT	Ureteral lesion	19
2	24	34w1d	2	*Percreta*	502	1432	-	Bladder wall lesion	6
3	25	31w3d	1	*Percreta*	278	1738	-	-	3
4	37	34w5d	1	*Increta*	3384	2967	-	Vaginal cuff lesion	7
5	33	38w2d	3	*Accreta*	1189	2128	-	-	6
6	39	36w	3	*Percreta*	512	1620	Gluteal necrosis	-	5
7	41	37w1d	0	*Increta*	1234	1436	-	-	3
8	32	25w6d	1	*Percreta*	1401	2049	-	Fetal death	14
9	28	33w2d	3	*Percreta*	754	1405	-	SWI	22
10	27	33w4d	4	*Increta*	1402	2559	AAT	-	4
11	25	36w2d	1	*Percreta*	1416	1304	-	-	8
12	34	35w4d	4	*Accreta*	484	778	-	-	10
13	40	33w4d	2	*Percreta*	563	2078	-	SWI	19
14	37	36w5d	2	*Increta*	633	1182	-	-	4
15	30	35w6d	3	*Increta*	512	978	-	-	5
16	35	33w3d	1	*Increta*	496	1192	-	-	3
17	32	33w3d	4	*Percreta*	803	1320	-	-	13
18	35	33w3d	1	*Accreta*	0	707	-	-	3
19	31	34w2d	2	*Percreta*	0	858	-	-	6
20	33	33w1d	4	*Accreta*	0	433	-	-	11
21	39	35w6d	1	*Accreta*	0	157	Gluteal skin lesion	-	3
22	29	36w	1	*Increta*	0	1293	-	-	2
23	38	33w3d	4	*Increta*	0	817	-	SWI and ureteral lesion	7
24	32	34w3d	3	*Accreta*	0	1006	-	-	13
25	36	29w6d	1	*Percreta*	0	1233	-	-	10
26	34	32w5d	2	*Percreta*	0	388	-	-	4
27	37	34w4d	1	*Increta*	0	448	-	-	7
28	30	34w4d	4	*Percreta*	0	917	-	-	8
29	35	36w5d	1	*Increta*	0	393	-	-	4
30	28	33w5d	2	*Accreta*	0	220	-	-	5
31	39	33w6d	1	*Accreta*	0	664	-	-	6
32	24	33w	2	*Percreta*	0	748	-	-	5
33	43	32w6d	2	*Percreta*	0	1469	-	-	18
34	40	35w2d	2	*Percreta*	0	1457	-	-	4
35	32	32w4d	0	*Accreta*	0	266	-	-	3

CBL = calculated blood loss; RBCs = red blood cells; AAT = acute arterial thrombosis; and SWI = surgical wound infection.

**Table 2:** Procedure time, blood transfusion, blood loss and hospital stay in each histological subgroup.

Histological degree	Number of cases	Mean procedure time*	Mean blood transfusions	Mean blood loss (mL)	Hospital stay (days)
*Accreta*	9	327 min	185.9 mL/0.78 U	706.5	6.7
*Increta*	11	321 min	834.0 mL/3.27 U	1,398.4	5.9
*Percreta*	15	342 min	415.3 mL/1.67 U	1,334.4	9.7
**Total**	**35**	**332 min**	**487.9 mL/1.94 U**	**1,193.0 mL**	**7.7**

* = Time from the beginning of anesthesia to the end of the abdominal wall closure.

**Table 3:** Mean cumulative blood loss volume from all endovascular procedures.*

Technique	Number of studies	Mean (range) cumulative blood loss in the intervention group (mL)
PBOAA	7	865.5 (613.6 to 1117.4)
PBOCIA	4	1650 (827.5 to 2473)
PBOIIA	25	1263 (95% CI 1030 to 1497.5)
PBOUA	3	1141 (265.3 to 2016.8)
UAE	7	2273.4 (980.5 to 3566.4)
**Current (PBOIIA + UAE)**	**-**	**1193 (157 to 2967; SD 679)**

PBOAA: prophylactic balloon occlusion of the abdominal aorta; PBOCIA: prophylactic balloon occlusion of the common iliac arteries; PBOIIA: prophylactic balloon occlusion of the internal iliac arteries; PBOUA: prophylactic balloon occlusion of the uterine arteries; UAE: uterine arteries embolization; mL: milliliters.

* extracted and adapted from Shahin and Pang, 2018 (13).
